# Halloysite nanotubes-enhanced epoxy acrylate latex emulsion as a novel anticorrosive protective coating for metal surface in 3.5% NaCl solution

**DOI:** 10.3389/fchem.2024.1325354

**Published:** 2024-03-07

**Authors:** Muhammad Asif, Matloob Ahmad, Muhammad Jawwad Saif, Muhammad Naveed Anjum, Magdi E. A. Zaki

**Affiliations:** ^1^ Department of Applied Chemistry, Government College University Faisalabad, Faisalabad, Pakistan; ^2^ Department of Chemistry, Government College University Faisalabad, Faisalabad, Pakistan; ^3^ Department of Chemistry, College of Science, Imam Mohammad Ibn Saud Islamic University (IMSIU), Riyadh, Saudi Arabia

**Keywords:** epoxy acrylate, halloysite nanotubes, coatings, corrosion resistance, metal protection

## Abstract

Corrosion is a major problem that can lead to the degradation of metal structures. In this study, we developed a novel corrosion-protective coating for metal substrates based on a modified epoxy acrylate formulation reinforced with halloysite nanotubes (HNTs). Epoxy acrylate oligomers were first synthesized through the acrylation of epoxy using acrylic acid, followed by copolymerization with butyl methacrylate/vinyl acetate monomers to produce grafted epoxy acrylates (GEA). HNTs were then incorporated into the polymeric dispersion at weight loadings of 1%, 1.5%, and 2%. The corrosion resistance and waterproofing properties of the coatings were evaluated. The results showed that steel samples coated with HNTs-modified GEA showed no signs of rusting even after 16 days of immersion in a corrosive solution, whereas those coated with GEA alone showed rusting after only 9 days. These results demonstrate the effectiveness of HNTs-modified GEA coatings in protecting steel surfaces against corrosion. The coatings are also water-resistant and can be easily applied. This work provides a new approach to developing corrosion-protective coatings for metal substrates.

## 1 Introduction

Corrosion of metal substrates is a persistent and costly problem in various industries, ranging from automotive and aerospace to infrastructure and marine applications. It leads to material degradation, structural damage, and economic losses. Therefore, the development of effective corrosion-resistant coatings is of great importance to mitigate the detrimental effects of corrosion and extend the service life of metal substrates (Jovičević-Klug and Rohwerder, 2023; Es-soufi et al., 2024). Carbon steel possesses significant potential in various industries due to several advantageous properties, including its low density, high resistance, and comparatively lower cost.

In recent years, polymer coatings have gained significant attention as promising corrosion protection solutions due to their ease of application, versatility, and ability to form protective barriers on metal surfaces. Among the different types of polymers, epoxy based polymers have been widely used in corrosion-resistant coatings due to their excellent adhesion, chemical resistance, and mechanical properties (Ou et al., 2021; Zhang et al., 2021; Randis et al., 2023). Extensive experimental studies are reported in literature citing the application of epoxy coatings on carbon steel surfaces as corrosion-resisting coatings (Abbout et al., 2021; El-Aouni et al., 2021; Hsissou et al., 2021; 2022). However, further enhancements are still desired to improve their corrosion resistance performance and durability, especially in harsh environments.

The predominant corrosion-resistant coatings in contemporary applications primarily consist of solvent-based formulations. However, over the past decade, waterborne coatings have garnered significant interest in the context of safeguarding metallic substrates. This heightened attention stems from the stringent environmental standards that mandate the minimization or regulation of volatile organic compound emissions to the most stringent levels achievable (Liu et al., 2016; Zhang et al., 2017). Liu and Wang reported notable improvements in the coatings’ barrier properties and corrosion resistance through the incorporation of graphene and dopamine-treated mesoporous-TiO_2_ particles, respectively (Liu et al., 2016; Wang et al., 2018). Tang introduced a self-curing waterborne epoxy resin coating with enhanced corrosion protection, particularly effective in harshly corrosive environments (Tang et al., 2019). Collectively, these studies underscore the potential to augment the corrosion resistance of waterborne epoxy coatings through diverse additives and modifications.

Nevertheless, the corrosion-resistant characteristics exhibited by waterborne coatings fall significantly short of those demonstrated by solvent-based coatings. This discrepancy arises due to the frequent retention of hydrophilic groups within the coating during the film formation process, thereby diminishing the coating’s efficacy in terms of vapor diffusion and moisture resistance. Consequently, the incorporation of corrosion inhibitors or fillers becomes imperative to enhance the corrosion resistance of waterborne coatings, with micro/nano-sized inorganic particles (referred to as inorganic additives) frequently employed for this purpose (Rahman et al., 2015; Das et al., 2017).

In this context, the incorporation of nanofillers, such as halloysite nanotubes (HNTs), into epoxy coatings has emerged as a promising approach to enhance their corrosion resistance properties (Moazeni et al., 2013; Feng and Cheng, 2016; Vijayan P et al., 2016; Asadi et al., 2019a; Asadi et al., 2019b; Yap et al., 2020; Tang et al., 2021).

HNTs are naturally occurring clay nanotubes with a unique hollow tubular structure, high aspect ratio, and excellent mechanical and thermal properties. These characteristics make HNTs attractive as reinforcing agents in polymer coatings, offering the potential to create a protective barrier against corrosive species, inhibit the diffusion of corrosive ions, and enhance the overall performance of the coating.

In this manuscript, we present the preparation and application of an epoxy acrylate latex emulsion containing halloysite nanotubes (HNTs) as a novel corrosion-resistant coating for carbon steel substrates. To the best of our knowledge, the utilization of this latex emulsion coupled with the subsequent integration of Halloysite Nanotubes (HNTs) for the purpose of enhancing corrosion resistance has not been documented in existing literature.

The findings of this study have the potential to contribute to the development of advanced corrosion-resistant coatings for various metal substrates. These coatings could have potential applications in diverse industrial sectors, such as the automotive, aerospace, and marine industries.

## 2 Materials and methods

### 2.1 Materials and chemicals

Araldite F, solvent-free and unmodified bisphenol- A epoxy resin was purchased from Huntsman group. Its epoxy contents are 5.20–5.35 Equiv/Kg (ISO 3001), having a density range from 1.15–1.20 g/cm^3^. Acrylic acid (AA, >99% purity) was supplied by power chemical industries (PVT) LTD. Pakistan. Benzimidazole (>99% purity) was purchased from Alfa Aesar (A Johnson Matthey Company) Karlsruhe Germany. Halloysite nanotubes (HNTs) were purchased from natural nano, United States of America. Potassium persulfate (KPS) and 2-hydroxy-2-methylpropiohenone (analytical grade) were used as a thermal curing agent and a photo-initiator. Deionized water (DDW) was used in all experiments. Potassium hydroxide (KOH, >99% purity) was used as a standard for titration (0.1 N). FTIR spectra were recorded on Perkin-Elmer Spectrum two spectrophotometer.

### 2.2 Experimental

#### 2.2.1 Synthesis of epoxy acrylate (EA) oligomer

A mixture containing a 2:1 M ratio of acrylic acid and Araldite F was taken in a four-neck reaction flask equipped with a nitrogen inlet, reflux condenser, and mechanical stirrer. The esterification reaction was carried out under optimized conditions at 90°C using benzimidazole (1phr) as a catalyst.

The progress of the reaction was monitored by titrating the reaction mass withdrawn after regular time intervals for unreacted acid until the vinyl ester resin of desired acid value (<5 mg KOH/g) was obtained. The sample taken from the reaction mixture was dissolved in ethyl alcohol (∼5 mL) for titration against standard KOH. Cooled the reaction mixture after achieving the desired acid value.

#### 2.2.2 Preparation of composite latex (GEA)

Deionized water (100 mL), potassium persulphate (0.1 g), and sodium dodecyl sulfate (SDS, 0.1 g) were taken in a four-neck reaction flask equipped with a mechanical stirrer, nitrogen inlet, and reflux condenser. At 80°C, butyl methacrylate monomer (2.0 g) was added dropwise to the reaction mixture for an hour duration. Subsequently, potassium persulphate (0.1 g) and epoxy acrylate (EA, 2.0 g) dissolved in acetone (10 mL) was added dropwise for the next hour. Stirring was continued at 80°C for 5 hours to obtain the desired emulsion.

#### 2.2.3 Preparation of HNT’s reinforced composite latex (HGEA)

HNTs with varying weight loading (0.5%, 1%, 1.5%, 2.0%, and 2.5%) were dispersed in deionized water under sonication for 30 min before proceeding with the previously described polymerization steps.

#### 2.2.4 Preparation of protective coating on steel

Separate trials were conducted for UV and thermal curing of protective coatings on steel plates. 2-Hydroxy-2-methylpropiophenone was used as a UV curing photo-initiator, whereas potassium persulphate was used as a thermal curing agent.

After the addition of desired curing agent, the mixtures were thoroughly mixed and degassed by microwave sonication for 20 min. The prepared emulsions were cast on the steel substrates as a thin film of thickness ∼ 300 μm at ambient temperature. The samples were cured by thermal treatment and UV irradiation according to the nature of the curing agent.

### 2.3 Characterization

#### 2.3.1 The extent of reaction (p)

The fraction of carboxyl groups that have reacted in time ‘t’ is called the extent of the reaction. The extent of the reaction was calculated from the acid values of the reaction mixture by using the formula given below.

p=1‐M/M0 (1)

M is the acid value of the reaction mixture at a time ‘t,’ and M0 is the acid value at time zero.

#### 2.3.2 FT-IR and morphological studies

FTIR spectra were obtained to identify the functional group changes. Scanning electron microscopy (SEM) was used to study the morphology of prepared coatings. The sample was sputter-coated with a thin layer of silver to make the surface conductive.

#### 2.3.3 Anticorrosion test

Electrochemical impedance spectroscopy (EIS) is employed with a CH1660E electrochemical workstation for corrosion analysis using a 3.5% NaCl solution. A standard three-electrode system was employed using coated samples as working electrodes, and polarization curves were recorded from −250 mV to +250 mV vs. open circuit potential at a 1 mV/s scan rate. EIS experiments covered frequencies from 0.01 Hz-10^5^ Hz at 0.01 V applied voltage. Testing occurred at 298 K.

Additionally, a 400-h salt spray test with 5% NaCl solution at 90% humidity, following ISO 9227–2012, assessed coating durability.

## 3 Results and discussion

### 3.1 Extent of reaction

Table 1 summarizes the compositions used to prepare different coatings.

**TABLE 1:** Sample code designation and corresponding composition of coatings.

Sample code	Epoxy acrylate (g)	BMA (g)	HNTs (%)
EA	1.85	0	0
GEA	1.85	3.2	0
HGEA-1	1.85	3.2	0.5
HGEA-2	1.85	3.2	1
HGEA-3	1.85	3.2	1.5
HGEA-4	1.85	3.2	2
HGEA-5	1.85	3.2	2.5

The reaction between diglycidyl ether of bisphenol A and acrylic acid was monitored by measuring acid values at regular time intervals. The acid value of the reaction samples decreased as the reaction between epoxy and acrylic acid proceeded. A decrease in acid value provides a sufficient clue of the reaction between acrylic acid and epoxy.

**FIGURE 1:**
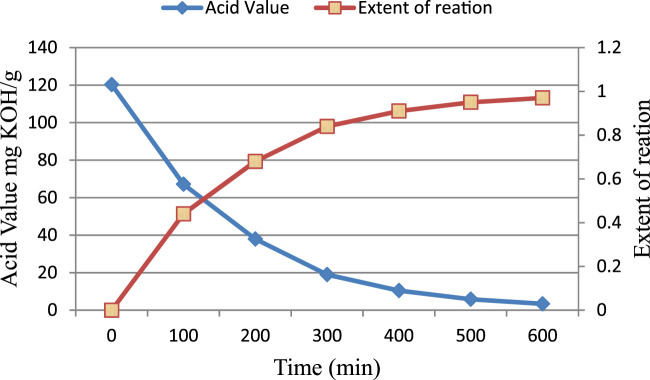
Acid value and extent of reaction *versus* time at 90°C.

**FIGURE 2:**
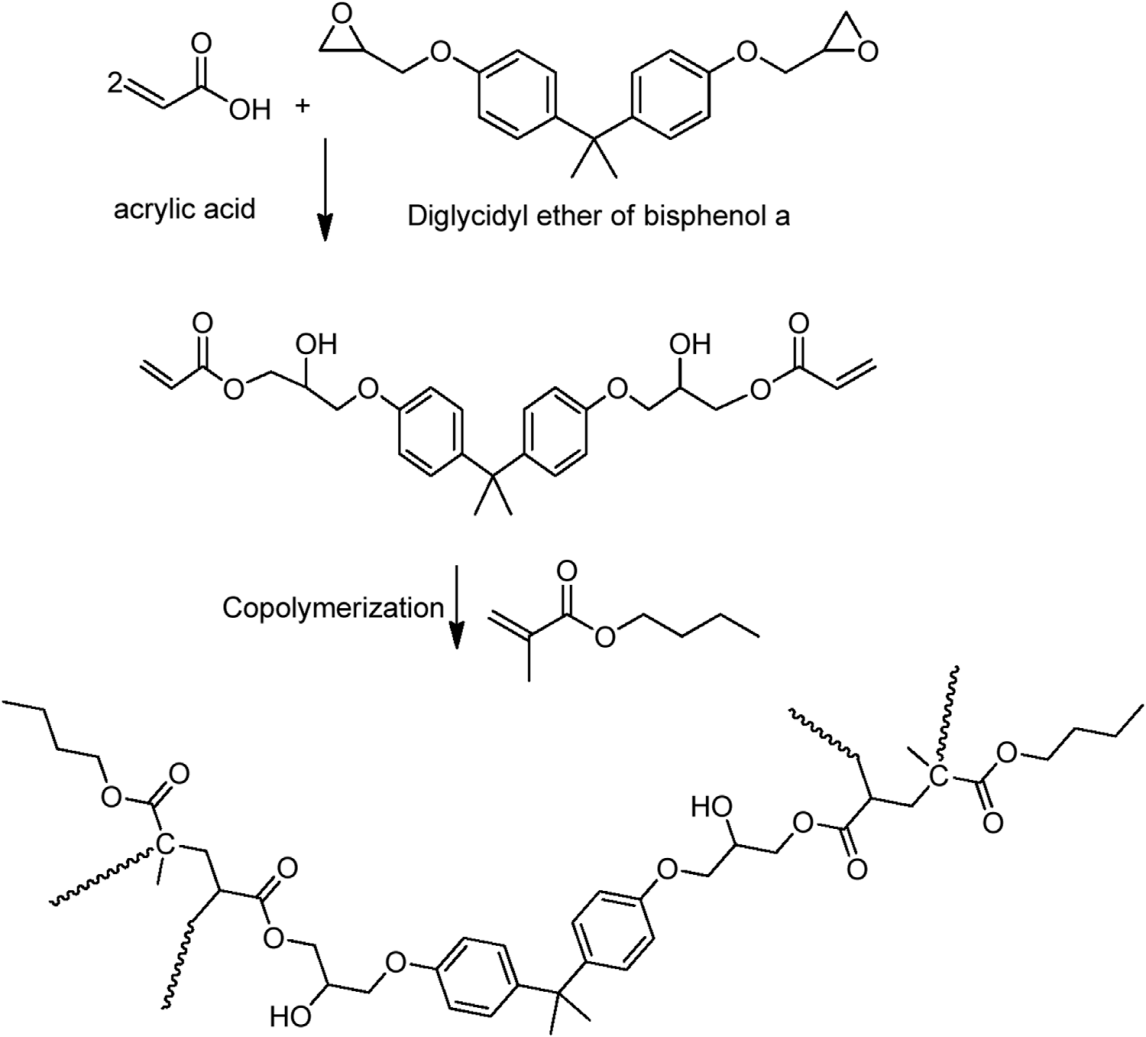
Preparation of epoxy acrylate resin and its copolymer with butyl methacrylate.

The initial rate of the reaction between acrylic acid and epoxy appears to be greater than the subsequent rate (Figure 1). The mixture became more viscous as the reaction progressed. An increase in viscosity may slow the reaction by limiting the effective collisions between the acrylic acid and epoxy species. The reaction between the epoxy group and acrylic acid causes the epoxy ring to open. The reaction is depicted schematically in Figure 2.

### 3.2 FTIR studies

**FIGURE 3:**
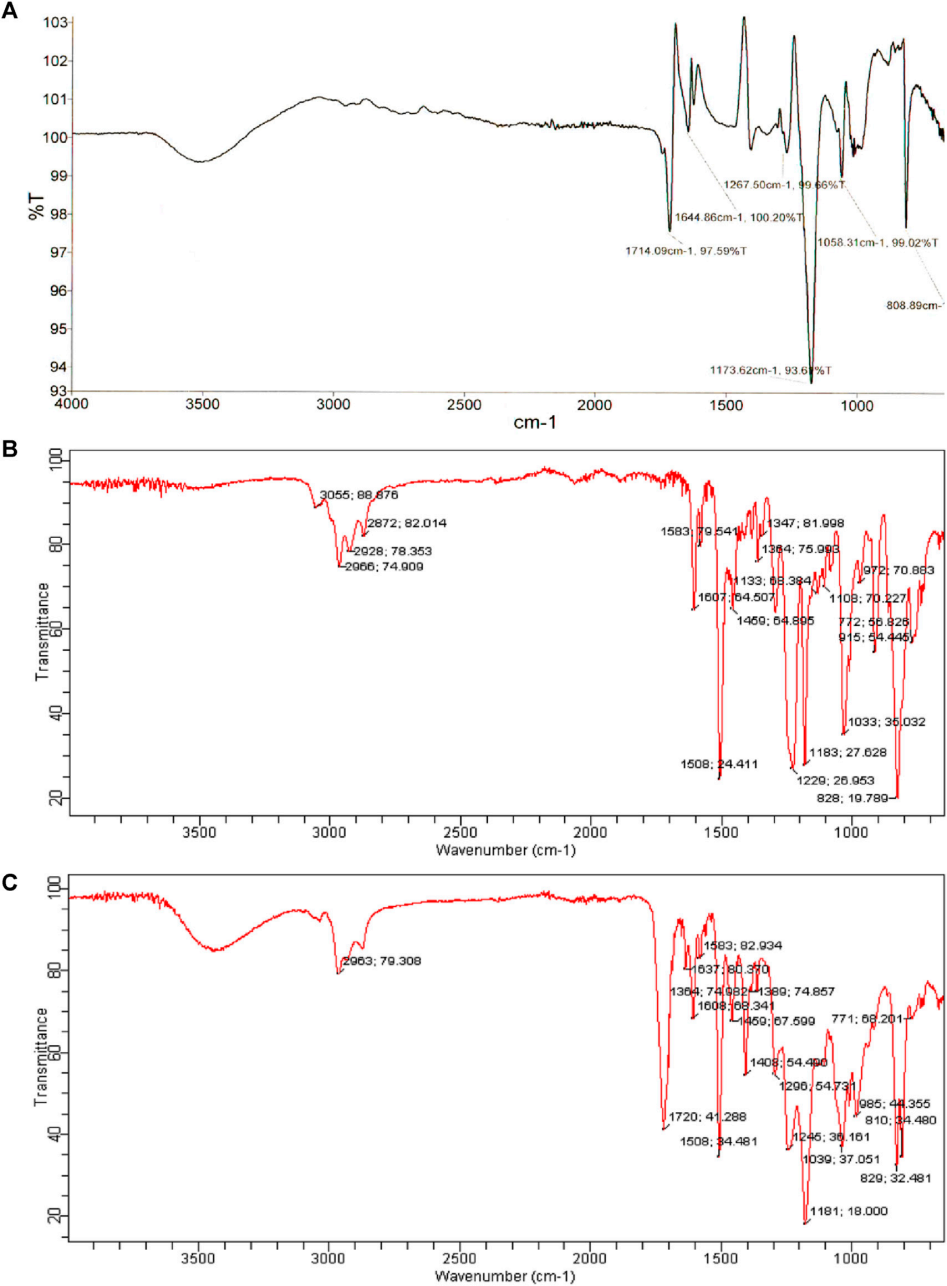
The FT-IR spectrum of **(A)** acrylic acid, **(B)** DGEBA epoxy resin and **(C)** synthesized vinyl ester resin.

Figure 3 shows the FTIR spectra of prepared samples. An FTIR analysis of the product showed the disappearance of the absorption peak for the oxirane group, which indicates the epoxy ring opening. The band at 1720 cm^-1^ is due to the carbonyl group of the ester formed. The acryloyl double bond attributed to a band at 1637 cm^-1^, indicated the formation of a vinyl ester. The band at 3300–3600 cm^-1^ in the vinyl ester spectrum is associated with the hydroxyl group with increased absorption intensity compared to the epoxy resin spectrum.

The epoxy groups have completely reacted, as indicated by the absence of an oxirane ring absorption band in the vinyl ester resin spectrum.

### 3.3 Latex (GEA) preparation

The presence of terminal vinyl groups on epoxy acrylate oligomer is an important feature used to further structural modifications. Emulsion polymerization of the epoxy-acrylate oligomer and butyl methacrylate produced an aqueous core-shell latex.

**FIGURE 4:**
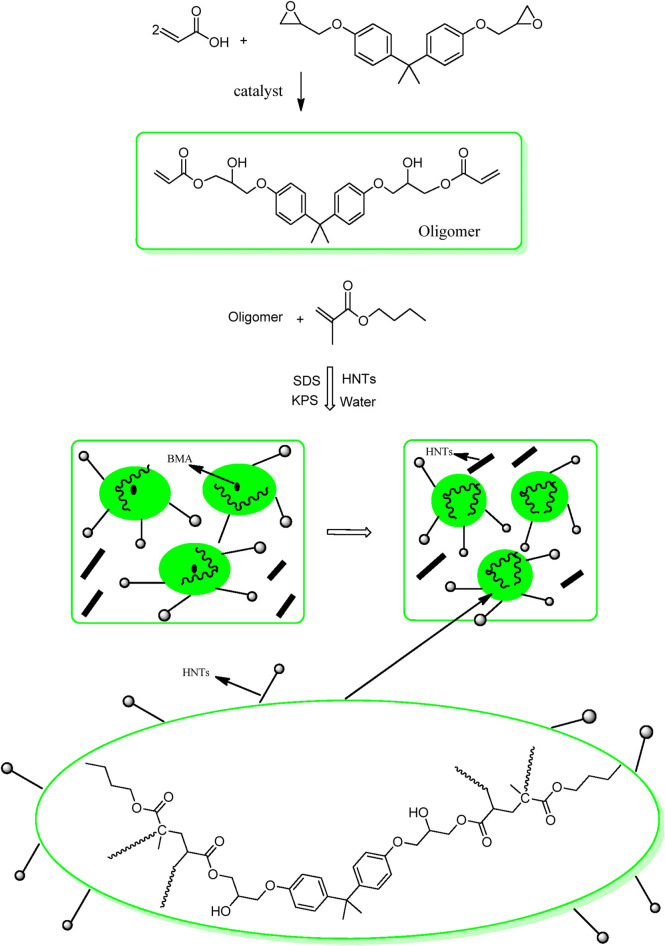
Scheme for preparation of modified epoxy-co-butyl methacrylate latex with HNTs.

The latex preparation is a consecutive two-step polymerization reaction. In the first step, butyl methacrylate (or vinyl acetate) is polymerized to form the core particles. Later, epoxy acrylate is introduced into the polymerization mixture to form the shell out of the core structure (Figure 4).

HNTs have hydroxy groups on their lumen structure and tend to agglomerate. Sonication was used to disperse these in the latex for 30 min before proceeding with the rest of the reaction.

Table 1 presents the sample codes and the corresponding compositions.

### 3.4 Film formation and adhesion characteristics

Epoxy resins exhibit superb adhesion to many substrates, good chemical and corrosion resistance, excellent electrical insulation, water resistance, and thermal stability. Coating applications require a high degree of adhesion, chemical, and corrosion resistance. The superb adhesion performance of epoxy resin is due to polar hydroxyl and ether groups lying at the backbone of epoxy resins (Lee et al., 1967; Caşcaval et al., 1997; Oprea et al., 2000). Most coating applications utilize acrylates due to better weather stability, gloss, and surface properties (Bajpai et al., 2002; Sharmin et al., 2004). Literature reports indicate that the strong interaction between epoxy coatings and the metal substrate can be attributed to both chemisorption and physisorption interactions. (El-Aouni et al., 2021).

Typically, acrylates cure via a mechanism involving free radicals and result in three-dimensional networks. Epoxy acrylates and epoxy novolac are widely used due to their superior adhesion, hardness, chemical resistance, and non-yellowing properties. Mechanical and thermal properties of cured epoxy resins are determined by the chemical and structural nature of the epoxy resins and crosslinker, the type, the concentration of cure initiators, cross-linking reaction conditions, epoxy conversion, and crosslinker to epoxy acrylate ratio.

The prepared HGEAs coatings showed excellent adhesion to steel plates. Improved cross-linking of different polymer chains with each other enhances the desirable characteristics. But if the cross-link density of polymeric chains is too high, then the coatings become too hard, and fragile leading to poor adhesion to steel substrates. Generally, conventionally recognized inorganic materials contain some hydroxyl groups on their surfaces due to the impact of the moisture present in the atmosphere (Tang et al., 2006). Possibly strong hydrogen bonding can occur between hydroxyl groups on the metal substrates and oxygen present in the polymer chains. Consequently, the hydrogen bonding between the oxygen of polymer chains and hydroxyl groups present on metal substrates significantly improved the adhesion of coatings on steel substrates.

### 3.5 Curing behavior of coatings

UV and thermal curing characteristics are described in Table 2. It is observed that below a 5% concentration of photo-initiator (2-hydroxy-2-methylpropiohenone) resulted in incomplete curing showing wet or tacky coatings even after exposure to UV-radiation of the intensity of 2 × 80 W/cm for more than 2 h. However, a 5% photo-initiator concentration resulted in the non-tackiness of coating films. After 90 min of UV curing, all the film mixtures were still sticky, and after 120 min, all the mixtures were no longer tacky.

**TABLE 2:** Film characteristics of modified-epoxy-co-polymer coating using 5% of the photo-initiator.

Sample code	UV-curing			Thermal curing		
	Exposure time (60 min)	Exposure time (90 min)	Exposure time (120 min)	Exposure time (60 min)	Exposure time (90 min)	Exposure time (170 min)
EA	Tacky	Slight tacky	Non-tacky	Tacky	Tacky	Non-tacky
GEA	Tacky	Slight tacky	Non-tacky	Tacky	Tacky	Non-tacky
HGEA-1	Tacky	Tacky	Non-tacky	Tacky	Slight Tacky	Non-tacky
HGEA-2	Tacky	Tacky	Non-tacky	Tacky	Tacky	Slight Tacky
HGEA-3	Tacky	Tacky	Non-tacky	Tacky	Slight Tacky	Non-tacky
HGEA-4	Tacky	Tacky	Non-Tacky	Tacky	Tacky	Non-tacky
HGEA-5	Tacky	Tacky	Non-Tacky	Tacky	Tacky	Non-tacky

In the thermal curing method (potassium persulphate as thermal curing agent), all samples were non-tacky at a temperature of 140°C for 170 min of exposure to heat.

### 3.6 Properties of cured films

The non-tacky drying of the films was observed for all compositions containing 5% photo-initiator and for thermal curing at 140°C for 170 min. All cured films showed an overall good performance.

The gloss of the films was excellent, and all films showed good water, acid, and alkali resistance. These tests were performed in 7 days immersion of sample in water, acidic and alkaline solution. 10% hydrochloric acid and 10% sodium hydroxide solutions were used to check the acid and alkali resistance of the coatings. The coatings were intact from any degradation after 7 days.

The water resistance behavior of coatings was studied by using contact angle measurements. The water contact angles for GEA coatings and HNTs-modified HGEAs coatings were 34.51° and 66.71°, respectively. The contact angle depends on the surface morphology; apart from the nature of the components, the hydrophilicity of coatings is reduced by incorporating HNTs in epoxy coatings. The surface roughness of HNTs-based coatings is greater than that of epoxy coatings. The increased surface roughness of the HNT-loaded coating could lower hydrophilicity and improve its resistance to water, acids, and alkalis. It has been observed that with an increasing proportion of halloysite, there is an enhancement in resistance to water, acids, and alkalis.

Another study has also reported halloysite nanotubes as capable of forming a protective barrier that effectively hinders the ingress of corrosive agents, thereby affording active corrosion protection to carbon steel. Furthermore, this barrier serves as an impediment to water infiltration within the coating (Liu and Li, 2023).

### 3.7 Thermogravimetric analysis

The thermal stability of the coating material plays a pivotal role, with its characteristics significantly shaped by factors such as network structure, chemical composition, type and concentration of residual polar groups, cohesive energy among molecular chains, molecular chain rigidity, various interaction parameters, and other chemical structural aspects, including steric strain and conformational arrangements of functional groups, among others. Thermogravimetric analysis of the various coating materials was performed to evaluate the thermal stability of the coating material.

**FIGURE 5:**
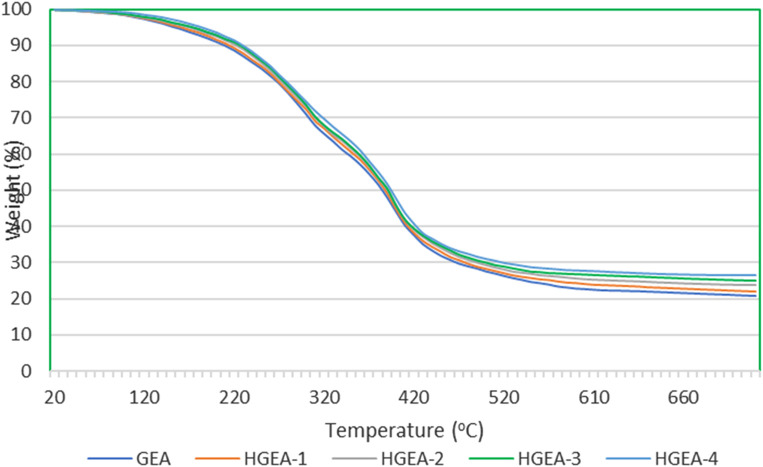
Thermogravimetric charts of the coating samples.

Figure 5 shows the TGA plots for selective samples. The nanocomposite with HNTs shows an increase in thermal stability with the increase in filler content.

The initial phase of degradation occurs up to 150°C, primarily ascribed to the evaporation of residual solvents and the release of entrapped moisture within the coatings. This stage is marked by a gradual weight loss of approximately 4% up to 150°C, followed by a more pronounced weight reduction of 50% within the temperature interval of 151°C–400°C.

The principal decomposition of the samples transpires during the second stage of decomposition, occurring above 150°C. This rapid decomposition process involves thermal cracking, gasification, disproportionation processes, and secondary dehydrogenation reactions.

### 3.8 Anticorrosion test

**FIGURE 6:**
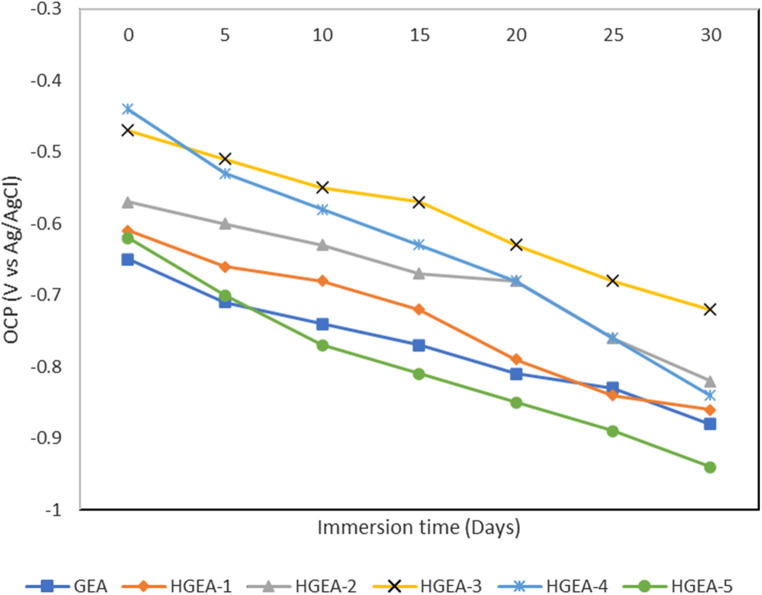
Open circuit potential (OCP).

The corrosion resistance of GEA and HNTs modified GEA coatings were evaluated by the electrochemical impedance spectroscopy. The corrosion resistance behavior of the different coating samplse immersed in 3.5% NaCl solution was studied. Initially, the open circuit voltage of GEA coating and HNTs modified GEA coating (HGEA-3) are approximately −0.65 and −0.47 V, respectively. As expected, the values of both the coatings started to decrease overtime gradually indicating the penetration of the corrosive solution through the coating. After 30 days, there is significant difference in the OCP values of both coatings where the HNTs modified GEA coating showed the better resilience (Figure 6). We do not see a clear correlation between HNTs loading and the corrosion resistance ability, but a 1.5% loading provided the optimum results.

**FIGURE 7:**
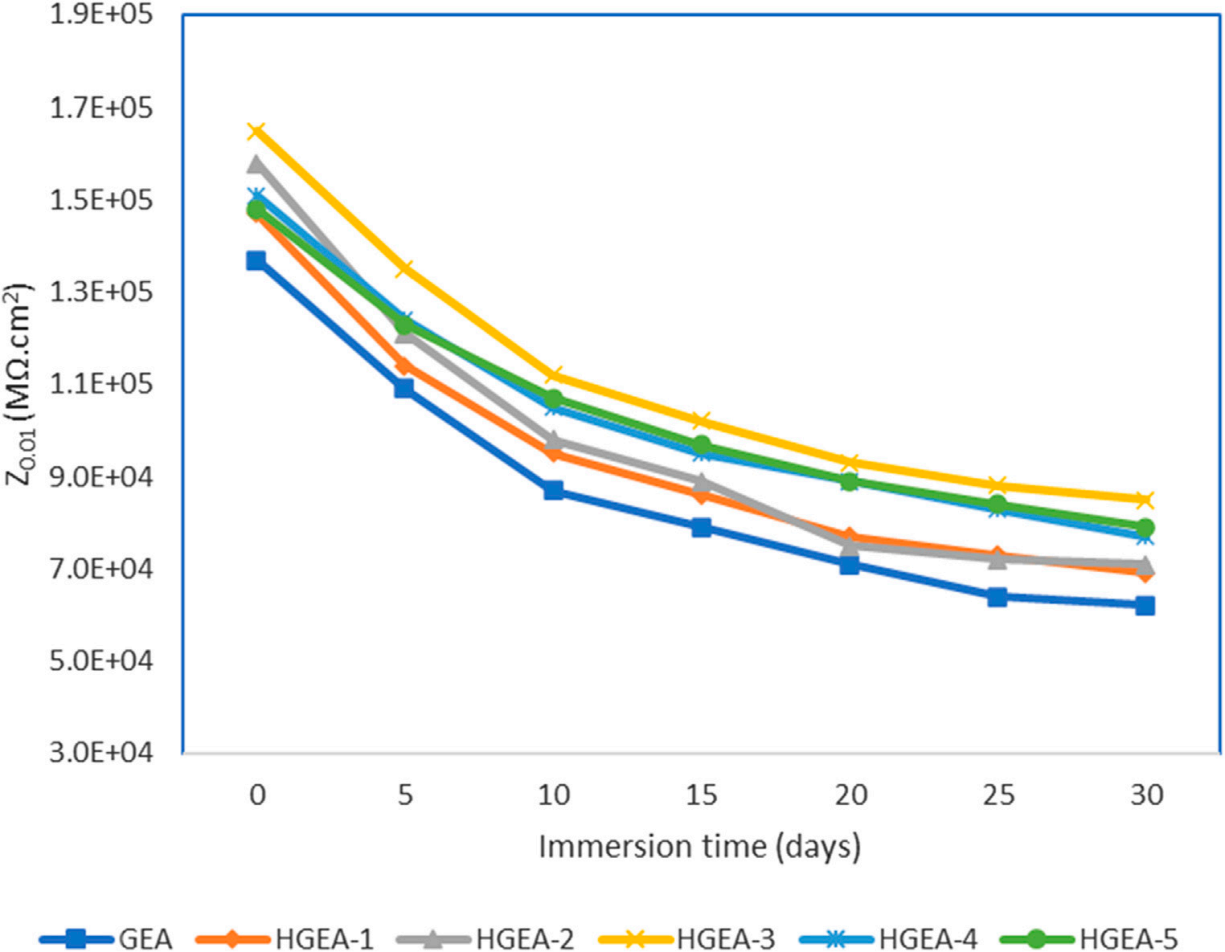
Impedance modulus (|Z|0.01 Hz) at frequency of 0.01 Hz at different intervals of immersion.

We have used the lowest frequency impedance modulus (Z_0.01 Hz_) as a quantitative indicator of corrosion resistance properties (Figure 7). Again GEA coating loaded with 1.5% HNTs provided the optimum results. This analysis additionally demonstrates that there is not a substantial disparity in the corrosion protection efficacy between GEA and HGEAs. Nonetheless, it is evident that HNTs have enhanced the corrosion protection capabilities of the coating to some extent.

**FIGURE 8:**
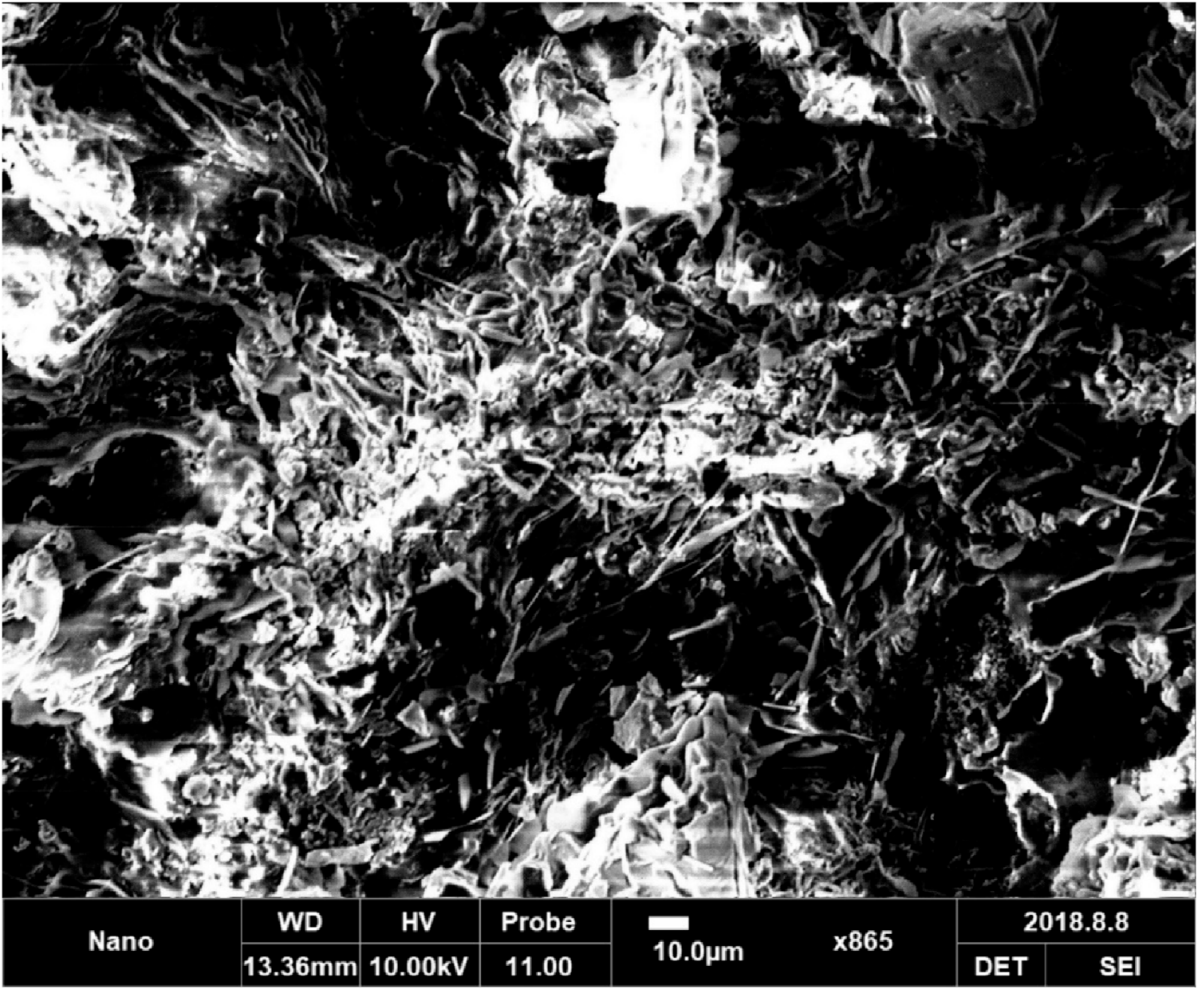
SEM images of cracked coating on steel substrate.

To assess the visual impact of the coatings, samples coated with GEA and GEA modified with HNTs were subjected to immersion in a 5% NaCl solution for a duration of 400 h. Rusting of the coating, particularly on the scratch edges, was visible in the GEA epoxy coatings (Figure 8). However, after salt immersion, the HNTs modified coatings remained intact. After 400 h (16 days) of immersion, the texture of the layers has slightly faded, but the coating has not been attacked by corrosion.

Samples with varying quantities of HNTs were tested. It was found that a 1.5% HNTs loading provided the optimum loading to achieve the best anti-corrosion results. The HNTs-modified epoxy coating has a lower void formation rate. Its film aids in keeping the bulk coating intact for a longer period. Furthermore, the degradation on the HGEA coating surface is uniform. Thus, the ability of HNTs to provide long-term coating sustainability is speculated. These results are in line with the EIS findings.

### 3.9 Surface morphology

**FIGURE 9:**
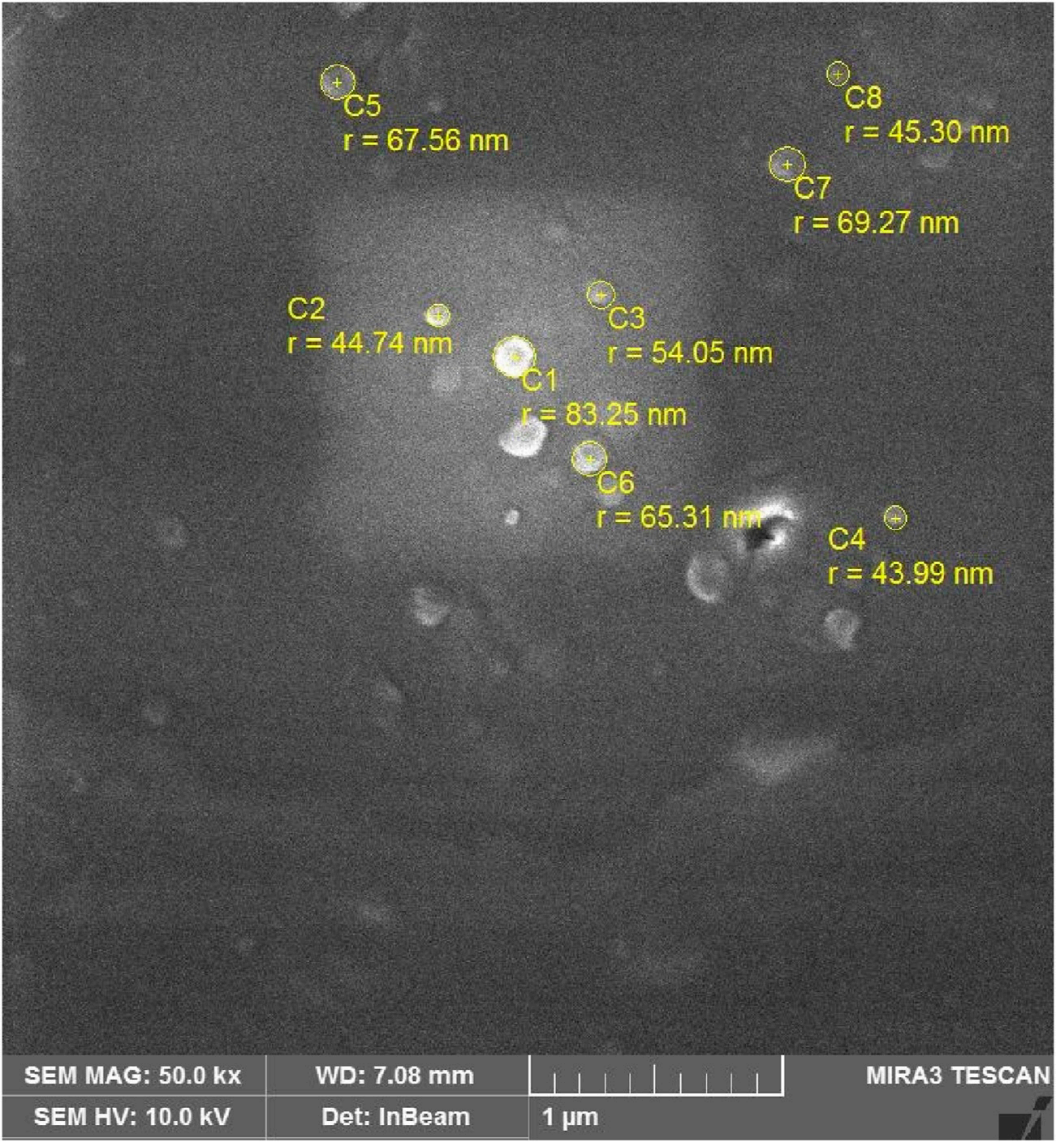
SEM images of halloysite reinforced epoxy acrylate nanocomposite.

HNTs are characterized by a diameter of less than 100 nm. Figure 9 shows the scanning electron microscopic image of a nanocomposite. The micrographs show fine dispersion of HNTs distributed in a regular pattern all around the matrix. The fine dispersion of HNTs in nanocomposites is due to mechanical and ultrasonic dispersion treatments. Furthermore, the epoxy acrylate matrix leaches into the lumen of nanotubes, improving interfacial interactions and bonding.

## 4 Conclusion

Our concluding observations and remarks are as follows:

1) We have developed and explored a modified epoxy acrylate coating using halloysite nanotubes (HNTs) additives as a corrosion protection coating for mild steel.
2) The latex preparation is a consecutive two-step polymerization reaction. In the first step, butyl methacrylate is polymerized to form the core particles. Later, epoxy acrylate is introduced into the polymerization mixture to form the shell out of the core structure. As a film-reinforcing material, 1%, 1.5%, and 2% weight loadings of HNTs are added to the polymeric dispersion.
3) GEA coatings provide suitable corrosion protection but GEA coatings with added HNTs demonstrated an enhanced corrosion protection ability.

Drawing upon the aforementioned observations, it is evident that the material under consideration exhibits exceptional suitability for coating metal surfaces to mitigate corrosion.

## Data Availability

The original contributions presented in the study are included in the article/Supplementary material, further inquiries can be directed to the corresponding authors.
